# Moderation effect of emotion regulation on the relationship between social anxiety, drinking motives and alcohol related problems among university students^*^

**DOI:** 10.1186/s12889-020-08776-5

**Published:** 2020-05-18

**Authors:** Sojung Kim, Jung-Hye Kwon

**Affiliations:** 1 Department of Psychology, Korea University, 145 Anam-Ro Seongbuk-Gu, Seoul, 02841 Republic of Korea; 2 Department of Psychiatry, Hanyang University Seoul Hospital, 222-1 Wangsimni-Ro Seongdong-Gu, Seoul, 04763 Republic of Korea

**Keywords:** Social anxiety, Alcohol related problems, Coping and conformity motives, Difficulties in emotion regulation, Moderated mediation modelling

## Abstract

**Background:**

Accumulated evidence suggests that individuals with social anxiety disorder (SAD) are at particular risk of developing alcohol use disorder (AUD). Yet, little is known about the mechanisms under this high comorbidity. This study aimed to elucidate the process of the development of alcohol related problems among individuals with elevated social anxiety. We investigated the moderation effect of difficulties in emotion regulation on the relationship between symptoms of social anxiety, coping and conformity motives and alcohol related problems.

**Methods:**

In a sample of university students (*N* = 647) in South Korea, we examined whether cognitive (fear of negative evaluation), behavioral (social avoidance), and physiological symptoms (concerns over physiological symptoms) of social anxiety affect alcohol related problems with the mediation of coping and conformity motives. Furthermore, difficulties in emotion regulation were hypothesized to moderate each mediational path.

**Results:**

Results showed that the fear of negative evaluation and concerns over physiological symptoms were associated with alcohol related problems with the mediation of conformity and coping motives, respectively. As hypothesized, each path was moderated by difficulties in emotion regulation.

**Conclusions:**

Findings suggest that coping and conformity motives to cope with cognitive and physiological symptoms of social anxiety were related to alcohol related problems. In addition, individuals with high levels of difficulties in emotion regulation were prone to exhibit more alcohol related problems.

## Background

### Alcohol related problems among individuals with social anxiety disorder: mediation of clinical characteristics of social anxiety and coping and conformity motives

Individuals with social anxiety disorder (SAD) are at a higher risk for alcohol or substance use disorders, even when compared to those with other anxiety disorders [1, 2]. This high rate of comorbidity is significant as it aggravates the daily functioning of individuals with SAD and can make the disorder chronic [3]. Over the last two decades, there have been a number of studies on alcohol related problems among individuals with SAD. Despite the frequent co-occurrence of the two disorders, inconsistent findings make it difficult to clarify the relationship between social anxiety and alcohol related problems. In previous research, social anxiety had negative associations with the frequency of drinking and binge drinking. In addition, social anxiety did not show a significant relationship with drinking quantity [4, 5]. Nevertheless, consistent findings show that SAD is positively related to alcohol related problems [6]. Emerging data suggest that this may be due to context-dependent drinking among individuals with SAD [7, 8] and it is different from drinking in individuals with typical alcohol use disorder (AUD). Therefore, it is necessary to investigate SAD-specific risk factors for alcohol related problems.

Buckner, Heimberg, Ecker, and Vinci [9] proposed a biopsychosocial model of social anxiety and substance use. According to their model, social anxiety-specific symptoms, such as physiological arousal, fear of negative evaluation, low positive emotion, perceived social deficits, and social avoidance, predicted dependence on substances via the motivation to cope with each symptom, which ultimately led to substance use disorders. While the biopsychosocial model [9] was the first to propose specific physiological, cognitive, and behavioral symptoms of SAD that contribute to the development of substance use disorders, it was a theoretical model for substance use disorders in general and did not conceptualize specific drinking motives related to each social anxiety symptom.

In previous research on alcohol related problems in SAD, individuals with elevated social anxiety drink to cope with their negative emotions (coping motives) or to conform as a means of getting along with others and ensure that they are not isolated (conformity motives) [10–12]. Moreover, accumulating evidence suggests that certain SAD symptoms, such as social anxiety or fear of evaluation, with the mediation of coping and conformity motives, are associated with drinking situations and alcohol related problems [13–16]. Considering that drinking with coping and conformity motives differs from socially acceptable drinking and is strongly related to alcohol related problems, even when drinking quantity is controlled [17], it is not surprising that people with SAD become dependent on it to regulate their symptoms and therefore experience more alcohol related problems, even if they drink a small amount of alcohol [10]. Despite these findings, insufficient research has been conducted to provide a comprehensive examination of the associations between each SAD symptom and alcohol related problems and to clarify the drinking motives involved.

### Impact of difficulties in emotion regulation on alcohol related problems in individuals with social anxiety disorder

When individuals with high social anxiety experience greater coping and conformity motives, they become more vulnerable to alcohol related problems. In addition, difficulties in emotion regulation strengthen the relationship between SAD and coping and conformity motives and increase the risk of alcohol related problems. Difficulties in emotion regulation refer to processes that influence the occurrence, intensity, duration, and expression of emotions [18]. It is strongly related to anxiety and depression and constitutes a risk factor for anxiety disorders and alcohol related problems [19]. Individuals with SAD experience not only anxiety in social situations but also chronically elevated negative emotions and dampened positive emotions in daily life [20]. The urgent need to resolve emotional distress increases the tendency to use maladaptive emotion regulation strategies, such as suppression, denial, rumination and drinking which provide instant satisfaction, rather than adaptive emotion regulation strategies, such as cognitive re-appraisal or acceptance, which require considerable cognitive resources in stressful situations [18]. This is risky as maladaptive emotion regulation strategies make individuals with SAD experience higher levels of negative emotions for a longer period of time and lead to secondary drinking problems [21–24]. Difficulties in emotion regulation are also associated with alcohol related problems. Difficulties in emotion regulation showed correlations with quantity and frequency of drinking and it was a variable that distinguished social drinking from alcohol dependence [25]. Application of maladaptive emotion regulation strategies showed a significant relationship with alcohol related problems [26].

Difficulties in emotion regulation is one of the cardinal factors in the development of AUD among individuals with anxiety disorders, as substance use disorders and emotional disorders share failure of negative emotion regulation as their diagnostic characteristics [27, 28]. In precedent studies, difficulties in emotion regulation mediated the relationship between negative affect which constitutes a dispositional characteristic of anxiety disorders, and drinking motives used to cope with this affect [29]. In addition, difficulties in emotion regulation moderated the relationship between anxiety sensitivity and alcohol related problems [30]. From a developmental perspective, young people with emotional disorders, including SAD, are at high risk of AUD [3, 31]. Drinking for the purpose of emotion regulation was identified as the most common cause of dependent drinking in the internalizing paths of AUD [32]. In particular, social anxiety among university students may be important as university is an environment high in social demands.

In light of these findings, emotion regulation appears to play a very important role in the developmental paths from social anxiety to alcohol related problems. Yet, to our knowledge, there is a dearth of empirical research on the role of coping and conformity motives and difficulties in emotion regulation in the relationship between SAD-specific symptoms and alcohol related problems. Hence, this study aimed to test a model in which cognitive-behavioral-physiological symptoms of social anxiety predict alcohol related problems with the mediation of coping and conformity motives, based on the biopsychosocial model of Buckner et al. [9]. We included fear of negative evaluation as the cognitive symptom, social avoidance as the behavioral symptom and concerns over physiological symptoms as the physiological symptom in the model, respectively. Also, we presupposed alcohol related problems with dependent drinking patterns, in accordance with previous findings that individuals with SAD indicate context-dependent drinking problems regardless of drinking quantity [4–6]. Furthermore, we hypothesized the moderation effect of difficulties in emotion regulation. That is, among individuals with elevated social anxiety, those with greater difficulties in emotion regulation were predicted to experience stronger coping and conformity motives, leading to an increase in alcohol related problems.

## Methods

### Participants and procedures

Participants were recruited nation-wide from multiple universities in South Korea. Of the 668 students who completed the survey, 21 were excluded due to the questionable validity of their responses (e.g. omitting more than 20% of the items, not providing demographic information). The final sample of 647 consisted of 445 females (68.8%) and 202 males (31.2%). The mean age was 20.92 (*SD =* 3.20) and the age ranged between 17 and 48 (Table 1). Participants were either introduced to this study during psychology classes or given information on the college community web page. All participants were informed that the purpose of the study was to investigate the relationship between social anxiety and alcohol related problems. The participants were provided a packet of self-report measures which took approximately 30 min to complete. The Institutional Review Board of the authors’ university approved the study procedures and all participants provided written informed consent prior to data collection.

**Table 1:** Characteristics of participants (*N* = 647)

	*M* (*SD*) or n (%)
**Age**	20.92 (3.20)
**Female**	445 (68.8%)
**Social Anxiety Disorder**	
SIAS >40^a^	157 (24.3%)
SPS > 28^a^	147 (22.7%)
**Alcohol Use Disorder**	
AUDIT ≥12^b^	172 (22.6%)

*SIAS* Social Interaction Anxiety Scale, *SPS* Social Phobia Scale, *AUDIT* Alcohol Use Disorders Identification Test

^a^Clinical cut-off scores of the Korean version of the SIAS and the SPS (Kim et al., 2013 [33]), ^b^Clinical cut-off score of the Korean version of the AUDIT (Lee et al., 2000 [34])

### Measures

#### Social phobia scale (SPS)

SPS was developed as a companion scale with SIAS by Mattick and Clark [35]. It consists of 20 items, which measure fear of performance related situations such as eating with others or being a focus of attention. Internal consistency, reliability, and validity were good in previous studies [35, 36]. Internal consistency of the Korean version of SPS was Cronbach’s α = .94 [33]. Internal consistency in this study was α = .92.

#### Social interaction anxiety scale (SIAS)

The SIAS was developed as a companion scale with SPS by Mattick and Clark [35]. It consists of 20 items, which measure fear of social interactions including talking to two or more persons. The SIAS has high internal consistency, retest reliability and excellent validity in previous studies [35, 36]. The internal consistency of the Korean version of SIAS was α = .88 [33]. The internal consistency in the current sample was α = .93.

#### Brief-fear of negative evaluation scale (B-FNE)

This scale measures fear of being negatively evaluated by others. Watson and Friend [37] originally developed the FNE and Leary [38] selected items with correlation scores higher than .50 for the brief version of the scale. The original scale was made as a binary scale, but we used the translated version of Lee and Choi [39] which was modified as a Likert scale which ranges from 1 (never) to 5 (very much). The internal consistency reported by Lee and Choi [39] was α = .93. Internal consistency in the current sample was α = .85.

#### Anxiety sensitivity Index-3 (ASI-3)

Reiss, Peterson, Gursky, and McNally [40] developed the ASI to assess fear of anxiety symptoms. Taylor and Cox [41] extended the scale by deleting six items from the original scale and adding 26 items. Later, Taylor et al. [42] modified the extended version of the ASI and developed the ASI-3, which consists of 18 items on three subscales: physical, social and cognitive concerns. We used the social concerns subscale in the Korean version of the ASI-3 [43] to assess concerns over physiological symptoms related to social anxiety. The internal consistency reported by Lim and Kim [43] was α = .87. In this study, the internal consistency for the total of 18 items was α = .91 and the internal consistency for the social concerns subscale was α = .82.

#### Social phobia diagnostic questionnaire (SPDQ)

The SPDQ is a 25-item self-report measure developed by Newman, Kachin, Zuellig, Constantino, and Cashman-McGrath [44] to diagnose SAD. Among the 25 items, 16 items were designed to measure fear and avoidance in different social situations. We used the total score of avoidance of social situations as a social avoidance variable in the model. The internal consistencies of the original version of the SPDQ and the Korean version of SPDQ were α = .92 and α = .91, respectively [44, 45]. The internal consistency in the current sample was α = .95.

#### Drinking motives questionnaire-revised (DMQ-R)

DMQ-R is a 20-item self-report measure developed to assess reasons for drinking alcohol by Cooper [17]. There are four subscales in the scale: coping motives, social motives, enhancement motives and conformity motives. DMQ-R had good internal consistency [17]. We used the Korean version of the DMQ-R validated by Ha and Tak [46]. In our sample, internal consistency of the DMQ-R total items was *α* = .91, and those of conformity subscale and coping subscale were *α* = .68 and α = .89, respectively.

#### Difficulties in emotion regulation scale (DERS)

DERS is a 36-item self-report measure to assess the level of difficulties in emotion regulation [47]. In a validation study, internal consistency was α = .93 and 4-8 week test-retest reliability and validity were also good [47]. We used the Korean version of the DERS which was validated by Cho [48]. Internal consistency of the K-DERS ranged from *α* = .92 to *α* = .93 [48]. The internal consistency in the current sample was *α* = .93.

#### Alcohol use disorders identification test (AUDIT)

The AUDIT was developed to screen AUD and dangerous drinking by the World Health Organization in 1989 [49]. In Korea, Lee, Lee, Lee, Choi and Nam [34] translated and validated the Korean version of the AUDIT which had good reliability and validity. There are three subscales in the AUDIT: alcohol dependence, harmful drinking, and hazardous drinking. In this study, internal consistency for the total of the items was *α* = .85. Based on previous research outcomes that social anxiety is related to alcohol related problems rather than drinking quantity or frequencies [4, 5], we used the harmful drinking subscale which asks about abnormal drinking behaviors and alcohol dependence and the hazardous drinking subscale, which asks about psychological side effects and alcohol related problems. The alcohol dependence subscale was not included as it focuses on alcohol consumption. The internal consistencies for the two subscales were *α* = .59 and *α* = .72, respectively.

### Statistical analysis

Descriptive statistics on epidemiological characteristics and psychological variables were analyzed via the IBM SPSS 20. Moderated mediation analyses were executed on the IBM SPSS AMOS 21. The moderated mediation model is a combined model of a mediation model and a moderated model which presupposes mediational effect and examines the differing levels of the mediational effect with the level of a moderator [50]. According to the suggestions of Preacher, Rucker, and Hayes [51], mediation modeling, moderated modeling, and moderated mediation modeling were conducted in sequence.

**Fig. 1:**
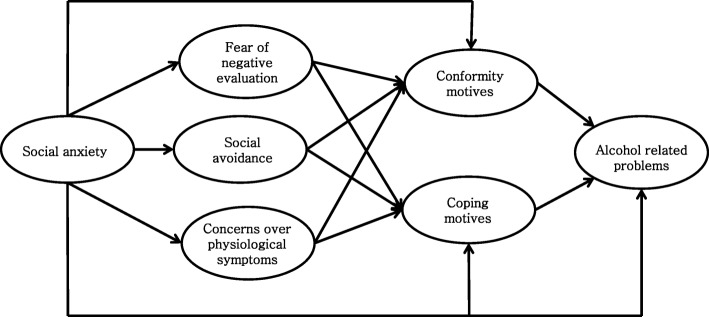
Cognitive-behavioral-physiological symptoms of social anxiety and drinking motives mediate the relationship between social anxiety and alcohol related problems

Firstly, to investigate the relationships between social anxiety symptoms, drinking motives and alcohol related problems, a mediational model was tested. We hypothesized that social anxiety would predict alcohol related problems via the cognitive (fear of negative evaluation), behavioral (social avoidance), and physiological symptoms (concerns over physiological symptoms) of social anxiety and drinking motives (coping and conformity motives). Therefore, mediational analysis was conducted using one independent variable (social anxiety), five mediators (fear of negative evaluation, social avoidance, concerns over physiological symptoms, conformity motives, and coping motives), and one dependent variable (alcohol related problems) (Fig. 1). Secondly, we tested the moderation effect of difficulties in emotion regulation in the relationship between social anxiety symptoms and drinking motives. Two separate moderation analyses were performed using one independent variable (fear of negative evaluation or concerns over physiological symptoms), one moderator (difficulties in emotion regulation) and one dependent variable (conformity motives or coping motives). Lastly, in our research model, a moderated mediation model was tested. Moderated mediation analysis was conducted using one independent variable (social anxiety), five mediators (fear of negative evaluation, social avoidance, concerns over physiological symptoms, conformity motives, and coping motives), one moderator (difficulties in emotion regulation) and one dependent variable (alcohol related problems). The number of bootstrap samples for bias corrected bootstrap confidence intervals was 10,000. For each model’s fit analysis, Tucker Lewis Index (TLI), Comparative Fit Index (CFI) and Root Mean Square Error of Approximation (RMSEA) were applied. In the analyses of data including more than 250 subjects, TLI and CFI are proper when they are bigger than .90, RMSEA is good if it is .05 or smaller, proper if .08 or smaller and not good if .10 or bigger [52, 53].

## Results

### Descriptive statistics and correlations between the variables

Before conducting structural equation modeling analyses, we administered descriptive statistics and correlation analysis between the variables.

The means of the SIAS and the SPS in this study (SIAS: *M* = 29.40, *SD* = 14.66; SPS: *M* = 19.22, *SD* = 13.72) were comparable to those of Korean young adult population (SIAS: *M* = 28.63, *SD* = 10.01; SPS: *M* = 19.51, *SD* = 12.15) [33]. Also, the mean of the AUDIT of this study (*M* = 8.39, *SD* = 5.88) was similar to that of Korean community population (*M* = 7.05, *SD* = 4.34) [34]. Among the participants, approximately 25% scored higher than the clinical cut-off scores of the Korean version of the SIAS and the SPS, respectively. Also, 22.6% of the total participants scored higher than the clinical cut-off score of the Korean version of the AUDIT (Table 1).

The SIAS and SPS had positive correlations with fear of negative evaluation, social avoidance, concerns over physiological symptoms and DERS. The SIAS and SPS were positively correlated with conformity motives and coping motives. However, SIAS showed a negative correlation with the total score of the AUDIT. SPS did not show a significant relationship with the total score of the AUDIT, *r* = .01, *p* = .86. The SIAS did not show a significant correlation with alcohol related problems, *r* = .03, *p* = .49, while the SPS had a positive correlation with alcohol related problems. The fear of negative evaluation, social avoidance, concerns over physiological symptoms and DERS had positive correlations with conformity motives and coping motives. Alcohol related problems did not show significant correlations with either fear of negative evaluation or social avoidance, all *ps* > .05. While concerns over physiological symptoms and DERS showed positive correlations with alcohol related problems.

Before we began the analysis of structural equation modeling, skew and kurtosis of each variable were inspected to test the normality. Results revealed that all variables were normally distributed (skew ≤3.0; kurtosis ≤10) [54]. Refer to Table 2.

**Table 2:** Descriptive statistics and correlations of the variables

	SIAS	SPS	FNE	Social avoidance	Concerns over physiological symptoms	DERS	DMQ-R	Conformity motives	Coping motives	AUDIT	Alcohol related problems
**SIAS**	1										
**SPS**	.76^**^	1									
**FNE**	.65^**^	.64^**^	1								
**Social avoidance**	.73^**^	.63^**^	.49^**^	1							
**Concerns over physiological symptoms**	.56^**^	.57^**^	.61^**^	.48^**^	1						
**DERS**	.55^**^	.46^**^	.50^**^	.47^**^	.45^**^	1					
**DMQ-R**	.06	.14^**^	.20^**^	.06	.22^**^	.22^**^	1				
**Conformity motives**	.22^**^	.22^**^	.29^**^	.16^**^	.25^**^	.27^**^	.56^**^	1			
**Coping motives**	.13^**^	.15^**^	.21^**^	.11^**^	.26^**^	.32^**^	.66^**^	.27^**^	1		
**AUDIT**	−.08^*^	.01	−.01	−.08^*^	.06	.07	.48^**^	.24^**^	.35^**^	1	
**Alcohol related problems**	.03	.11^**^	.06	.02	.15^**^	.17^**^	.39^**^	.25^**^	.35^**^	.90^**^	1
***M***	29.40	19.22	35.49	20.54	7.02	81.01	46.01	4.51	7.83	8.39	2.81
***SD***	14.66	13.72	8.32	10.45	5.08	20.15	14.21	2.04	3.82	5.88	3.70
**Skew**	0.29	0.91	−0.15	0.29	−0.03	0.51	0.21	1.65	1.03	1.00	2.03
**Kurtosis**	−0.48	0.82	−0.57	− 0.42	0.71	0.20	−0.14	2.56	0.53	1.35	5.27

*SIAS* Social Interaction Anxiety Scale, *SPS* Social Phobia Scale, *FNE* Fear of Negative Evaluation Scale, *DERS* Difficulties in Emotion Regulation Scale, *DMQ-R* Drinking Motives Questionnaire-Revised, *AUDIT* Alcohol Use Disorders Identification Test

^*^p < .05. ^**^p < .01

### Relationship between social anxiety and alcohol related problems: Mediational effect of cognitive-behavioral-physiological characteristics of social anxiety and coping and conformity motives moderated by difficulties in emotion regulation

We examine the moderated mediational effects of the clinical characteristics of social anxiety and coping and conformity motives in the relationship between social anxiety and alcohol related problems, based on the biopsychosocial model of SAD and substance use disorders [9] (Buckner et al., 2013). In our research model, the clinical characteristics of social anxiety: fear of negative evaluation, social avoidance and concerns over physiological symptoms were hypothesized to predict alcohol related problems with the mediation of coping and conformity motives. Furthermore, we supposed that difficulties in emotion regulation would moderate the relationship between cognitive-behavioral-physiological characteristics of social anxiety and coping and conformity motives.

### Mediation model analysis

Among the research variables in the study, the fear of negative evaluation, social avoidance, and difficulties in emotion regulation were made into latent variables via item parcelling [55].

**Fig. 2:**
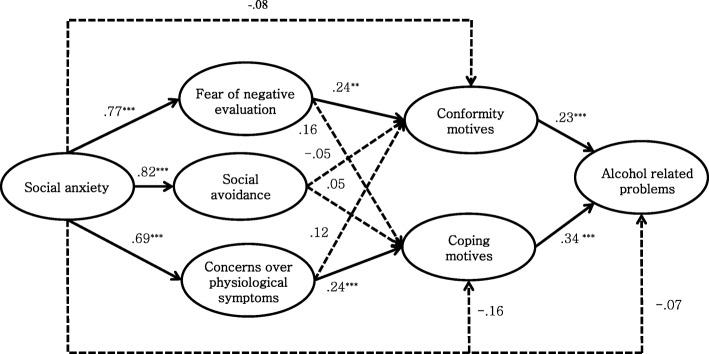
Mediation model of clinical characteristics of social anxiety and coping and conformity motives. **p < .01. ***p < .001

Results from the mediation model analysis showed that the research model fits our data well, *χ*^2^ (234, *N* = 647) = 581.313, TLI = .953, CFI = .960, RMSEA = .048 (90% CI = .043–.053). Social anxiety was positively correlated with the fear of negative evaluation, social avoidance and concerns over physiological symptoms. The fear of negative evaluation predicted conformity motives, while the path from fear of negative evaluation to coping motives was not significant. Social avoidance did not significantly predict conformity motives and coping motives. Concerns over physiological symptoms positively predicted coping motives. However, it did not significantly predict conformity motives. Both conformity motives and coping motives positively predicted alcohol related problems. Meanwhile, a direct path from social anxiety to alcohol related problems was not significant. In addition, direct paths from social anxiety to conformity motives and coping motives were not significant, respectively. For the details, refer to Fig. 2.

Considered collectively, results from mediation analysis suggest that social anxiety does not directly predict alcohol related problems, but it indirectly predicts alcohol related problems. Social anxiety predicts coping and conformity motives via the mediation of cognitive and physiological symptoms. In turn, cognitive and physiological symptoms of social anxiety predict alcohol related problems with the mediation of coping and conformity motives.

To examine the significance of indirect effects of social anxiety, bootstrapping was conducted according to the suggestions of Shrout and Bolger [56]. The indirect effects were estimated from 10,000 samples that were randomly drawn from the raw data and were tested in the 95% confidence interval. Results showed that direct effect of social anxiety on alcohol related problems was not significant, while the indirect effect was significant. The indirect effect of concerns over physiological symptoms on alcohol related problems was significant. Also, the indirect effect of fear of negative evaluation on alcohol related problems was significant. However, social avoidance did not show a significant direct effect on either conformity motives or coping motives. It also did not show a significant indirect effect on alcohol related problems. Direct, indirect and total effects are presented in Table 3.

**Table 3:** Direct, indirect and total effects between variables in the mediation model

Path	Direct effect	Indirect effect (confidence Interval)	Total effect
Social anxiety → Social avoidance	.82^***^	–	.82^***^
Social anxiety → Concerns over physiological symptoms	.69^***^	–	.69^***^
Social anxiety → Fear of negative evaluation	.77^***^	–	.77^***^
Social anxiety → Coping motives	−.16	.33(.05–.63)	.17^**^
Social anxiety → Conformity motives	.08	.23(−.08–.53)	.31^***^
Social avoidance → Coping motives	.05	–	.05
Social avoidance → Conformity motives	−.05	–	−.05
Concerns over physiological symptoms → Coping motives	.24^**^	–	.24^**^
Concerns over physiological symptoms → Conformity motives	.12	–	.12
Fear of negative evaluation → Coping motives	.16	–	.16
Fear of negative evaluation → Conformity motives	.24^*^	–	.24^*^
Social anxiety → Alcohol related problems	−.07	.13(.07–.20)	.06
Social avoidance → Alcohol related problems	–	.01(−.11–.11)	.01
Concerns over physiological symptoms → Alcohol related problems	–	.11(.04–.20)	.11^**^
Fear of negative evaluation → Alcohol related problems	–	.11(.01–.22)	.11^*^
Coping motives → Alcohol related problems	.34^***^	–	.34^***^
Conformity motives → Alcohol related problems	.23^**^	–	.23^**^

^*^p < .05. ^**^p < .01. ^***^p < .001

### Moderated model analysis

We tested moderated models under the suggestions of Preacher et al. [51]. We examined the moderation effect of difficulties in emotion regulation on 1) the path between fear of negative evaluation and conformity motives and 2) the path between concerns over physiological symptoms and coping motives. Ping’s method [57] was applied to test the significance of the model fit and moderation effects.

**Fig. 3:**
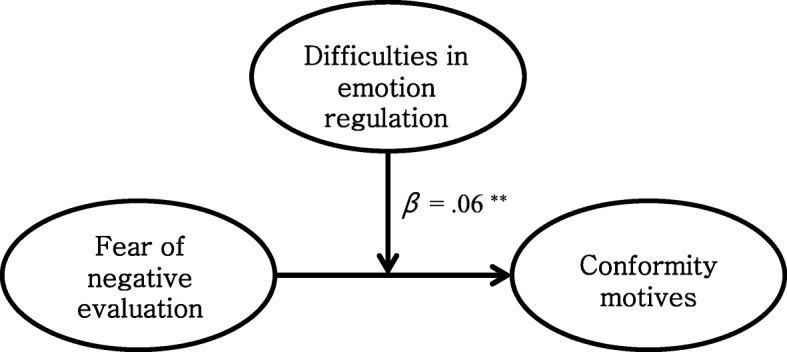
Difficulties in emotion regulation moderate the relationship between fear of negative evaluation and conformity motives. **p < .01

**Fig. 4:**
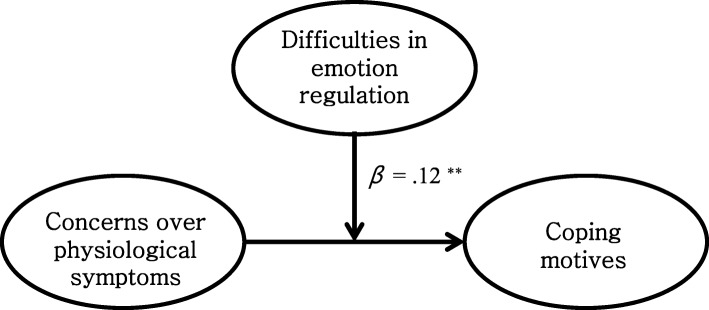
Difficulties in emotion regulation moderate the relationship between concerns over physiological symptoms and coping motives. **p < .01

Results showed a good model fit to the data. The model fit of the moderation model of difficulties in emotion regulation on the relationship between fear of negative evaluation and conformity motives (moderation model 1) was good, *χ*^*2*^ (39, *N* = 647) = 102.999, TLI = .977, CFI = .984, RMSEA = .050 (90% CI = .039–.062). A direct path from the interaction variable (fear of negative evaluation x difficulties in emotion regulation) to conformity motives was also significant, *β* = .06, *p <* .01. In addition, the moderation model of difficulties in emotion regulation on the relationship between concerns over physiological symptoms and coping motives (moderation model 2) fits the data well, *χ*^*2*^ (35, *N* = 647) = 266.024, TLI = .959, CFI = .967, RMSEA = .057 (90% CI = .050–.065). Also, a direct path from the interaction variable (concerns over physiological symptoms x difficulties in emotion regulation) to coping motives was significant, *β* = .12, *p <* .01. Refer to Figs. 3 and 4.

**Fig. 5:**
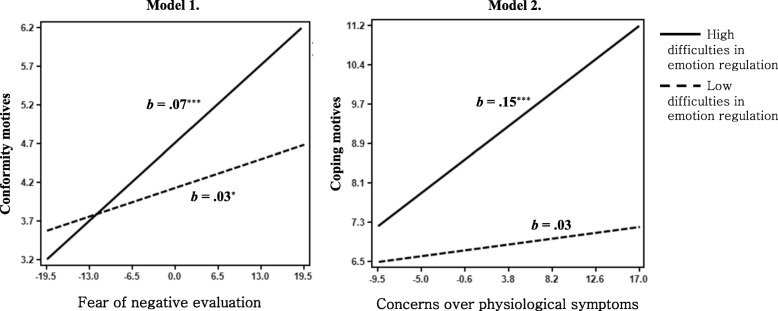
Moderation effect of difficulties in emotion regulation in the relationship between social anxiety specific symptoms and coping and conformity motives. Model 1. Moderation effect of difficulties in emotion regulation in the relationship between fear of negative evaluation and conformity motives. Model 2. Moderation effect of difficulties in emotion regulation in the relationship between concerns over physiological symptoms and coping motives. *p < .05. ***p < .001

To further examine the moderation effect of difficulties in emotion regulation, we divided participants into two groups: a high difficulties in emotion regulation group (+ 1 SD above the mean) and a low difficulties in emotion regulation group (− 1 SD below the mean). Results supported that the level of difficulties in emotion regulation moderated the effect of fear of negative evaluation on conformity motives and the effect of concerns over physiological symptoms on coping motives, respectively. See Fig. 5.

The high difficulties in emotion regulation group showed a larger moderation effect in the relationship between the fear of negative evaluation and conformity motives compared to the low difficulties in emotion regulation group, respectively, *b* = .07, *p <* .001, *b* = .03, *p <* .05. Also, the high difficulties in emotion regulation group showed a significant relationship between concerns over physiological symptoms and coping motives, *b* = .15, *p <* .001. While the low difficulties in emotion regulation group did not show a significant relationship between concerns over physiological symptoms and coping motives, *b* = .03, *p* > .05.

### Moderated mediation model analysis

**Fig. 6:**
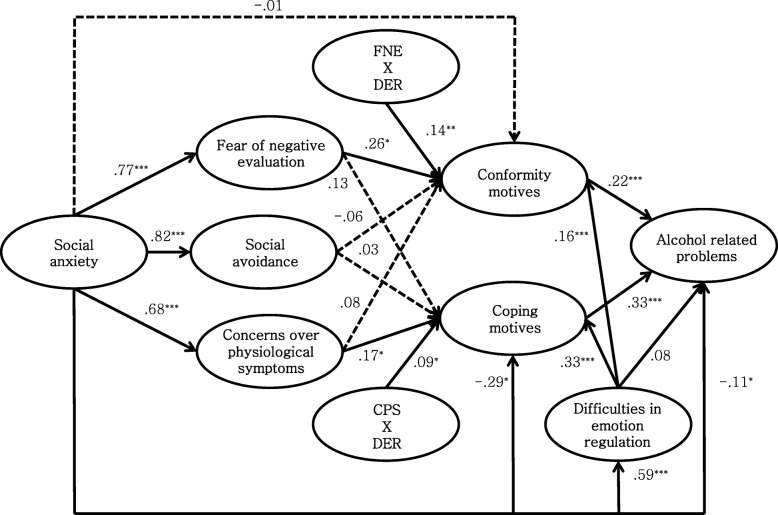
Results from examination of the moderated mediation model. Note. FNE: Fear of negative evaluation; CPS: Concerns over physiological symptoms; DER: Difficulties in emotion regulation. *p < .05. **p < .01. ***p < .001

Lastly, we analyzed a moderated mediation model that included two interaction terms (fear of negative evaluation x difficulties in emotion regulation, concerns over physiological symptoms x difficulties in emotion regulation). The moderated mediation model fits the data well, *χ*^*2*^ (370, *N* = 647) = 1247.499, TLI = .913, CFI = .926, RMSEA = .061(90% CI = .057–.064). See Fig. 6 for details.

We examined whether difficulties in emotion regulation moderate 1) the indirect effect of fear of negative evaluation on alcohol related problems via conformity motives, 2) the indirect effect of concerns over physiological symptoms on alcohol related problems via coping motives when using the bootstrapping method. Results showed that the indirect path from the interaction term of fear of negative evaluation and difficulties in emotion regulation to alcohol related problems with the mediation of conformity motives was significant. Also, the indirect path from the interaction term of concerns over physiological symptoms and difficulties in emotion regulation to alcohol related problems with the mediation of coping motives was significant. Refer to Table 4.

**Table 4:** Direct, indirect and total effects of variables in the moderated mediation model

Path	Direct Path	Indirect Path (Confidential Interval)	Total Effect
Social anxiety → Difficulties in emotion regulation	.59^***^	–	.59^***^
Social anxiety → Social avoidance	.82^***^	–	.82^***^
Social anxiety → Concerns over physiological symptoms	.68^***^	–	.68^***^
Social anxiety → Fear of negative evaluation	.77^***^	–	.77^***^
Social anxiety → Coping motives	−.29^*^	.44(−.18–.72)	.15^**^
Social anxiety → Conformity motives	−.01	.30(−.01–.61)	.29^***^
Difficulties in emotion regulation * Concerns over physiological symptoms → Coping motives	.09^*^	–	.09^*^
Fear of negative evaluation * Difficulties in emotion regulation → Conformity motives	.14^*^	–	.14^*^
Difficulties in emotion regulation → Coping motives	.33^***^	–	.33^***^
Difficulties in emotion regulation → Conformity motives	.16^*^	–	.16^*^
Social avoidance → Coping motives	.03	–	−.03
Social avoidance → Conformity motives	−.06	–	−.06
Concerns over physiological symptoms → Coping motives	.17^*^	–	.17^*^
Concerns over physiological symptoms → Conformity motives	.09	–	.09
Fear of negative evaluation → Coping motives	.13	–	.13
Fear of negative evaluation → Conformity motives	.26^*^	–	.26^*^
Social anxiety → Alcohol related problems	−.11	.16(.08–.25)	.05
Concerns over physiological symptoms * Difficulties in emotion regulation → Alcohol related problems	–	.03(.00–.07)	.03^*^
Fear of negative evaluation * Difficulties in emotion regulation → Alcohol related problems	–	.03(.01–.08)	.03^*^
Difficulties in emotion regulation → Alcohol related problems	.08	.14(.09–.21)	.22^**^
Social avoidance → Alcohol related problems	–	−.00(−.11–.09)	−.00
Concerns over physiological symptoms → Alcohol related problems	–	.08(.01–.16)	.08^*^
Fear of negative evaluation → Alcohol related problems	–	.10(.00–.21)	.10^*^
Coping motives → Alcohol related problems	.33^***^	–	.33^***^
Conformity motives → Alcohol related problems	.22^**^	–	.22^**^

^*^p < .05. ^**^p < .01. ^***^p < .001

## Discussion

This study examined a moderated mediation model in which the symptoms of social anxiety predict alcohol related problems via the mediation of coping and conformity motives and that the mediational paths were moderated by difficulties in emotion regulation. Results showed that the cognitive and physiological characteristics of social anxiety were directly and indirectly associated with alcohol related problems via the mediation of coping and conformity motives. In addition, difficulties in emotion regulation moderated the mediational paths for each symptom.

Of the cognitive characteristics of social anxiety, fear of negative evaluation predicted alcohol related problems through conformity motives. This finding is consistent with those of previous studies in which the fear of negative evaluation was the strongest predictor of alcohol related problems mediated by coping and conformity motives and was more strongly correlated with alcohol related problems relative to social anxiety or avoidance [58]. However, the path from fear of negative evaluation to alcohol related problems mediated by coping motives was not significant; this result was inconsistent with those of Stewart et al. [11], in which the fear of negative evaluation predicted alcohol related problems via the mediation of both coping and conformity motives. This difference may have occurred because the fear of negative evaluation was the only variable related to symptoms of SAD in the research model of Stewart et al. [10], while multiple mediators pertaining to the behavioral and physiological characteristics of social anxiety were included in the current study and revealed distinctive paths for each variable. In other words, our analyses confirm the theory that individuals who fear the evaluation of others in social situations experience problems with alcohol consumption because of drinking based on external motives to fit in and avoid being isolated. Further, this external and negative reinforcement is a more important motive than resolving their internal discomfort. Meanwhile, concerns over physiological symptoms predicted alcohol related problems via the mediation of coping motives. This fits the theory that socially anxious individuals use substances both to reduce uncomfortable sensations resulting from physiological arousal and to hide their symptoms from others [9]. In line with previous findings, concerns over physiological symptoms were related to conformity motives.

In a study conducted by Terlecki and Buckner [16], of the four drinking motives proposed by Cooper (1994) [17], coping and conformity motives exerted the strongest mediational impacts on drinking in SAD, and each motive was specifically correlated with symptoms of SAD. Their results showed that social anxiety predicted drinking in personal and intimate situations and those that increase negative emotions via the mediation of conformity and coping motives. Based on the findings, they discussed that conformity motives were related to drinking to fit in and to avoid negative evaluation and rejection [59, 60], and drinking because of coping motives could occur in personal and intimate situations leading to certain physiological SAD symptoms, or circumstances that increase negative emotions. The current study has significance as it extended the study of Terlecki and Buckner [16] to predict alcohol related problems by examining the clinical characteristics of social anxiety individually to verify whether the fear of negative evaluation mediated conformity motives and concerns over physiological symptoms mediated coping motives.

The paths from social avoidance, which is a behavioral characteristic of social anxiety, to conformity and coping motives were non-significant, and therefore, did not support the research hypothesis. Individuals with elevated social anxiety might experience negative reinforcement by drinking to participate in social situations, but individuals with high social avoidance might experience negative reinforcement through the instant reduction of tension concerning expected social situations by not participating in those situations at all. However, the finding indicating that there was no significant path between social avoidance and coping motives, which is related to solitary drinking with negative emotions, is believed to be related to the participants’ characteristics. Individuals with internalizing problems, who tend to drink alone and report serious depression and anxiety, show older age of onset [61, 62]. Participants of this study were young university students without pronounced functional impairment. Therefore, it is likely that they had not developed the habit of solitary drinking as a coping motive. Consequently, future research should target a wider age group and determine whether social avoidance is a protective factor in alcohol related problems [4] or exerts different effects on alcohol related problems according to age.

This study also examined whether, of individuals with elevated social anxiety, those with greater difficulties in emotion regulation experienced strong coping and conformity motives and if this increased alcohol related problems. Results demonstrated that difficulties in emotion regulation moderated the paths in which the fear of negative evaluation predicted conformity motives, and concerns over physiological symptoms predicted coping motives. Individuals who are unable to cope with the cognitive and physiological symptoms of social anxiety by using adaptive emotion regulation strategies could exhibit a tendency to cope via drinking, which could imply that they are vulnerable to alcohol related problems [63, 64]. Findings indicating that the impact of emotion regulation was important in the path that connected the cognitive and physiological characteristics of social anxiety to conformity and coping motives have clinical implications: training for emotion regulation focusing on each clinical characteristic of social anxiety could help to mitigate alcohol related problems. For example, cognitive and behavioral interventions for improving objective views and guided participations in social situations might be beneficial. In addition, interventions supporting people to experience the natural decreasing process of arousal through acceptance, rather than suppressing physiological symptoms or tension through drinking, could aid the prevention and treatment of alcohol related problems among individuals with elevated social anxiety.

## Limitations

The limitations of the study were as follows. The proposed model was tested with cross-sectional data from self-report measures. Considering that the dependent variable was alcohol related problems which is related to a behavioral aspect and that the purpose of the study was to explore the clinical characteristics that mediate alcohol related problems among individuals with elevated social anxiety, it could have limited the examination of the path connecting risk factors with problem behaviors using a one-time questionnaire [65]. In addition, the participants were university students, rather than clinical patients or a representative population sample. Therefore, it is difficult to consider that our data represents alcohol related problems among the entire group of individuals with SAD. However, early adulthood, including college years, is a high risk period for alcohol related problems. A prospective study found that more than a quarter of students with SAD developed AUD before they become 30 [31]. This study has implications in that it provides a glimpse of interactions between social anxiety-specific symptoms and drinking motives in predicting alcohol related problems in young individuals. To examine the paths from social anxiety to alcohol related problems with a focus on the clinical characteristics of SAD, future studies could benefit from including individuals diagnosed with SAD in a wide age range.

## Conclusions

Despite these limitations, as an empirical study, this study contributes to the literature that examines the integrated process from the cognitive and physiological symptoms of social anxiety to alcohol related problems via coping and conformity motives, moderated by difficulties in emotion regulation. Of the studies conducted to examine the causal mechanisms underlying alcohol related problems in SAD during the past two decades, few have verified a comprehensive model based on empirical data, and theoretical models requiring verification were proposed not long ago [9, 66, 67]. This study has significance, as it examined the mechanism underlying alcohol related problems in SAD by focusing on specific social anxiety symptoms and drinking motives via the modified biopsychosocial model of substance use disorder in SAD [9]. The results suggest that interventions targeting cognitive and physiological symptoms of social anxiety and emotion regulation training could prevent habitual drinking in stressful situations among individuals with elevated social anxiety. It will be important to continue to research alcohol related problems among individuals with SAD targeting social anxiety-specific patterns.

## Data Availability

The datasets used and/or analysed during the current study are available from the corresponding author on reasonable request.

## Abbreviations

AUD: Alcohol Use Disorder
SAD: Social Anxiety Disorder
